# ‘[*M*]y own pace and space, without the pressures’: Online learning experiences of audiology students

**DOI:** 10.4102/sajcd.v71i1.1012

**Published:** 2024-05-22

**Authors:** Liepollo Ntlhakana, Aadilah Alli

**Affiliations:** 1 Department of Audiology, Faculty of Humanities, University of the Witwatersrand, Johannesburg, South Africa

**Keywords:** online, learning, qualitative, audiology students, experiences, self-regulated, instructors

## Abstract

**Background:**

Online learning has been used to improve students’ learning experiences and to allow students to engage with their own learning in various spaces. However, there is a dearth of literature on students’ experiences with online learning in the field of audiology.

**Objectives:**

This study aimed to describe the conditions of online learning, explore the challenges and benefits of online learning and determine strategies that students employ while engaging with online learning.

**Method:**

An exploratory qualitative research design was employed. Audiology students from the second to the fourth year participated in the study. Qualitative data were collected online via MS Teams using a semi-structured interview schedule with the participants. Thematic analysis was used to analyse the participants’ interviews.

**Results:**

Most of our participants were females in their fourth year of study. The students accessed the online learning platforms procured by the university from their homes, with reported benefits such as the flexibility and independence of learning, and time and cost-effectiveness. However, challenges such as limited internet connectivity, issues with time management and inconsistent power supply restricted positive experiences with online learning.

**Conclusion:**

The online learning benefits that were reported by the students and the compensatory strategies they employed facilitated self-regulated learning. The study’s findings highlighted the need for continuous checking-in with students regarding their experiences with the learning approaches intended to improve engagement with their courses. These results could be used as a guide for curriculum planning that is student-focused.

**Contribution:**

Students’ experiences explored in our study provided a guide for online learning approaches that were suitable for audiology students. Student-centred and self-regulated learning practices were highlighted and future studies may further explore these frameworks and theories.

## Introduction

Online learning as a domain of learning has implications for students’ experiences and perceptions of learning in institutions of higher learning (IHL). Online learning can be defined as a method of education whereby students learn virtually through electronic technologies and media (Tamm, 2020). This learning approach was first introduced in the early 1990s during the early days of the internet and became most prevalent in IHL worldwide during coronavirus disease (COVID-19) pandemic hard lockdown (World Health Organization, 2020). During this period, a quick transition to emergency remote teaching (ERT) took place, and more than 80% of students in the Global North seamlessly continued with online learning with minor challenges, but the experiences of students in the Global South were different (Laher et al., 2021; Mupawose et al., 2021; Thome et al., 2020; UNESCO, 2020). The differences in students’ experiences of online learning highlighted possible restrictions that limited access to online learning for students in the Global South (Laher et al., 2021).

To prioritise access to online learning in South Africa, IHL introduced the new ERT, which was intended for continuous learning and teaching during the COVID-19 hard lockdown in 2020 (University of the Witwatersrand, 2020). However, this was met with some challenges such as limited time allocated to design courses and for students to access online courses (Rawashdeh et al., 2021; Rad et al., 2021), and acquaintance with using new learning management systems (LMS), which restricted the timeous adoption of online learning to both the students and the lecturers (Mpungose, 2020). In addition, limitations around infrastructure, for example, buildings that did not support online learning and the lack of consistent and reliable electricity supply affected other functions such as access to the internet and usability of training equipment, which in turn further exacerbated exclusions to online learning for students most notably for students in low-to-middle-income countries (LMICs) (Dube, 2020; Singh et al., 2022; Zarei & Mohammed, 2021; Mupawose et al., 2021). Additional challenges reported by students were around engagement with course content in the absence of course instructors during theoretical and clinical teaching (Kim et al., 2021; Laher et al., 2021). Although these had negative implications on students’ experiences with online learning, there is a dearth of knowledge specific to the students in the audiology programmes in South Africa, thus, online learning approaches that were student-centred and designed for students in LMICs should be reimagined continuously (Babu & Reddy, 2015; Khoza-Shangase et al., 2021).

Student-centred learning approaches promote integrated educational programmes intended to encourage students’ engagement with their own learning (Lee & Hannafin, 2016; Panadero, 2017). Previous studies have reported the successful application of the practices of online learning with students in healthcare professions such as speech therapy, audiology, dentistry, medicine and nursing programmes (Khoza-Shangase et al., 2021; Kim et al., 2021; Masuku & Mupawose, 2022); however, these studies were not foregrounded on the student-centred learning approaches. Ölçek et al. (2021) reported that some audiology students struggled to grasp knowledge of the theoretical courses in audiology that were offered online. Thus, Ölçek et al. (2021) suggested that the audiology department’s curriculum review should align the theory courses with the audiology practicum competency outcomes. However, audiology programmes in South Africa already follow a curriculum that was guided by competency outcomes as mandated by the Health Professions Council of South Africa (HPCSA, 2017). Therefore, it was imperative to contextualise competency-based outcomes through empirical evidence to determine indicators for student-centred learning suitable for audiology programmes in South Africa.

Therefore, a need to continuously explore research in online learning that was student-centred during and post-pandemic and specific to IHL for audiology programmes in South Africa was crucial. Students’ experiences and engagements with online courses and learning materials vary, thus, working within frameworks that were student-focused accommodated and motivated students to own their learning and encouraged self-directed learners (Bączek et al., 2021; Bharuthram & Kies, 2013; Laher et al., 2021; Winne & Marzouk, 2019). However, there was a dearth of evidence-based literature that simultaneously investigated students’ learning experiences and socio-emotional well-being during and post-pandemic. Hence, it was important to understand the students’ experiences that best supported online learning and were student-focused.

## Research methods and design

### Aim

This study aimed to explore the online learning experiences of audiology students at a South African university.

### Objectives

- To describe conditions influencing the online learning experiences of audiology students
- To explore the challenges and benefits of online learning that impact the experiences of audiology students
- To determine strategies that audiology students employed when engaging with online learning.

### Research design

An exploratory qualitative research design was employed to understand the experiences of audiology students regarding online learning. The participants’ features for students between the second and fourth years of study were described as guided by the research objectives and based on the study’s inclusion criteria (Leedy & Ormrod, 2021).

### Study setting

The study site was the Department of Speech Pathology and Audiology, Audiology Division, at the University of the Witwatersrand. This context was chosen because of its feasibility, convenience and easy access to participants at the University of the Witwatersrand. The audiology department (study site) employed online learning, which was readily phased in as per the university’s implementation teaching and learning plan for 2020–2024 (University of the Witwatersrand, 2020). Online platforms, including Microsoft Teams (MS Teams) and Big Blue Button (BBB), are LMS that have been used since 2020. Prior to the COVID-19 pandemic, audiology students used an LMS called Sakai and later the introduction into Ulwazi (canvas) in 2021. In 2021, the university introduced a blended learning approach and lecturers designed their blended learning courses as Ulwazi courses to ensure access to course materials, lecturers for consultations and course assessments.

### Participants

In this study, participants were selected using a purposive sampling method (Showkat & Parveen, 2017). Audiology students from the second to fourth year who were registered during the period from 2020 to 2022 were invited to participate in the study. The researchers followed two steps to reach the participants: (1) the administrator of the audiology department distributed the participant information sheet to the participants via email and (2) potential participants interested in participating in this study were asked to directly contact the researcher. Subsequently, the snowball sampling technique was used to attract additional participants (Naderifar et al., 2017). At the point of data saturation, a total of 10 (*n* = 10) participants were reached. Data saturation was determined by the repetitiveness of the participants’ responses and the researchers’ belief that after coding the eighth interview, no new codes emerged from additional interviews (Saunders et al., 2017).

Table 1 illustrates the participants’ demographic information. Nine of the 10 participants were females, with the age range between 19 and 29 years. The majority of our participants were in their fourth year of study, which is the final year of an undergraduate professional degree. All our participants were students who were commuting daily from home to the university.

**TABLE 1:** Participants’ demographic information.

Participant	Age (years)	Gender	Residence versus Home	Number of years studying Audiology at the University of the Witwatersrand	Current year of study	Number of years engaging with online learning at the University of the Witwatersrand
1	21	Female	Home	4	4	3
2	29	Female	Home	3	4	3
3	22	Female	Home	4	4	3
4	21	Female	Home	4	4	3
5	23	Female	Home	4	4	3
6	26	Female	Home	5	4	3
7	22	Female	Home	3	3	2
8	19	Female	Home	2	2	1
9	22	Male	Home	3	3	2
10	23	Female	Home	3	3	2

### Data collection

The data were collected using a self-designed interview guide (Appendix 1) that was informed by the literature and the study aim. This tool was pre-tested before being used in the main study. Semi-structured interviews were conducted with the 10 participants. These semi-structured interviews that lasted approximately 35 min, were conducted on Microsoft Teams. While six participants agreed to use the video option, four chose to keep their videos off during the interviews. The interviews were audio recorded using the recording feature on MS Teams, to allow the researcher to review and replay the interviews, transcribe data and identify key information during data analysis (Cook, n.d.). The researcher (A.A.) maintained a reflexive journal to document their position as an audiology student while collecting data for the study (Haynes, 2012).

### Data analysis

Interview transcriptions were analysed using thematic analysis, which facilitated the identification, organisation, and description of themes. These themes were deductively coded based on the main study aim (Bingham & Witkowski, 2021). This provided valuable insights into the students’ experiences, which were essential for the final interpretations of the study’s findings (Braun & Clarke, 2006). Finally, member checking was employed to ensure consistency, transparency and truthfulness of the data collected (Birt et al., 2016; Cypress, 2017; Schwandt et al., 2007).

### Ethical considerations

Before conducting data collection for the study, non-medical ethical clearance to conduct this study was obtained from the University of the Witwatersrand School of Human and Community Development Ethics Committee (no. STA_2022_23), and then participants were invited to participate. Participants who consented to participate in the study. During data collection, participants’ online interviews that were collected, were protected. In addition, access to interviews during data collection was controlled by using the waiting room feature to vet participants. The researcher (A.A.) employed the use of a reflexive journal to avoid interview bias (Haynes, 2012).

## Results

The results presented in this section addressed the research question, which was ‘What are the experiences of audiology students regarding online learning?’ Four themes emerged from the data analysis and pertained to the audiology students’ experiences regarding online learning. These themes were: (1) learning spaces and devices used for online learning, (2) benefits of online learning, (3) challenges with online learning and (4) strategies employed to facilitate online learning. The themes are detailed hereafter, and the sub-themes and interview excerpts from participants are presented in Table 1, Table 2, Table 3 and Table 4.

**TABLE 2:** Learning spaces and information communication technology used by participants for online learning.

Learning spaces and ICT	Interview excerpt
Home environment	‘I could work efficiently, no disturbances, everything was just okay. I think I couldn’t complain. It was just like a library on campus, if not better.’ (P4, female, 4th year student, engaged in online learning for 3 years) ‘I felt like I could work effectively at home. Just obviously I think being at home for too long is also not good because that’s when the lack of motivations would kick in because you just constantly always at home and it’s the same thing every day. But I felt most of the time I was working better at home.’ (P10, female, 3rd year student, engaged in online learning for 2 years)
Computer and Internet	‘I have my own personal computer. I have my own personal cell phone that I can refer to. So, with regards to online learning I was really privileged in the sense that I had all the resources available to me.’ (P1, female, 4th year student, engaged in online learning for 3 years) ‘I’ve got constant access to a computer and the Internet.’ (P2, female, 4th year student, engaged in online learning for 3 years)

ICT, information communication technology.

**TABLE 3:** Online learning benefits reportedly experienced.

Benefits of online learning	Interview excerpt
Time-and-cost effective	‘I didn’t really need to get up so early to come to [name of the South African University] for like an 8:00 AM lecture, which I definitely would have in first year because the schedule is so full. So, I think that also helped because I wasn’t doing quite as much driving and I had more time.’ (P7, female, 3rd year student, engaged in online learning for 2 years). ‘I think even though I was using my own data at home, it probably was more cost effective being online than coming to [*name of the South African University*]. I’m just thinking of petrol prices because it takes me a good hour every day to get varsity and to come back home. So, like 60 kilometers, that’s a tank, like that’s a lot of money.’ (P10, female, 3rd year student, engaged in online learning for 2 years)
Less exhausting	‘I was exhausted during the day, and with online learning I could get my 8 hours of sleep. There was enough time to study and to go through my work. So that was great … Traffic like going between [*name of the University*] and home was a couple of hours. So, I got to have my 8 hours of sleep. I got to wake up have a proper breakfast, do my studying and then when I wrote the paper, I felt prepared. I wasn’t tired.’ (P1, female, 4th year student, engaged in online learning for 3 years) ‘It’s sometimes nice to just have a bit of a break from the driving, so I can catch up on sleep and organize my day.’ (P7, female, 3rd year student, engaged in online learning for 2 years)
Flexible learning	‘I love to interact with people, no doubt I’m very social, but I personally prefer to learn in my own space at my own pace, without the pressures of a lecturer, supervisor, other people. I truly preferred and appreciated the opportunity to just work at my own pace. I think it removed a lot of anxiety.’ (P4, female, 4th year student, engaged in online learning for 3 years) ‘I could reorganize my own timetable and work in periods where I felt more productive as opposed to like being forced to work because it’s allocated in specific times and having to switch between subjects.’ (P9, male, 3rd year student, engaged in online learning for 2 years)
Independent learning	‘It definitely made me more independent instead of relying on the notes they give us and all the resources that they would give.’ (P3, female, 4th year student, engaged in online for 3 years) ‘I guess it teaches you self-dependence. You had to learn how to be your own lecturer, take it all in, understand everything by yourself, make sense of what you’re reading as opposed to listening.’ (P4, female, 4th year student, engaged in online for 3 years)

**TABLE 4:** Online learning challenges reportedly experienced.

Challenges with online learning	Interview excerpt
Difficulty managing time	‘I found it a bit challenging because I couldn’t manage my time properly and then I found myself listening to like 4 lectures before an exam or before a test.’ (P5, female, 4th year student, engaged in online for 3 years) ‘It has been really difficult for me to manage my time. I’m usually a very disciplined person, but because it was online and recorded as well, I took advantage of the whole situation, so time management has always been a problem.’ (P6, female, 4th year student, engaged in online for 3 years)
Limited instructor presence	‘I kind of felt like they took the easy way out and they made a video and uploaded it, and then they were like, oh, if you have any questions, we can do a session. But if you don’t have any questions, then we won’t even do a session and I’m like you supposed to teach us for seven hours a week while you’re now teaching us one hour a week. … The lecturer should do it as if it was in person, not just using last year’s lecture slides and uploading them even with the wrong dates on them for example. That is just, in my mind, ill prepared and quite insulting to the students as well that you didn’t even like bother to change the dates …’ (P2, female, 4th year student, engaged in online for 3 years) ‘Some aren’t great with replying to emails, so obviously interaction was limited in that regard and so often many people just would kind of withdraw from asking for help or from seeking help.’ (P4, female, 4th year student, engaged in online for 3 years)
Limited peer interaction	‘I wasn’t like fully able to get to know my class either. Like I would just see their name on the screen, and I don’t know them from like a bar soap.’ (P7, female, 3rd year student, engaged in online for 2 years) ‘I didn’t know the group that I was with well enough, you lost touch. So, the only thing we really had going for us was work, well course related stuff. So, you would only ask people about that. We didn’t get that much time to make proper friends.’ (P9, male, 3rd year student, engaged in online for 2 years)
Connectivity issues	‘When load shedding did struck, if my laptop died, I couldn’t do anything until it came back.’ (P1, female, 4th year student, engaged in online for 3 years) ‘The interruption with the connectivity, it made me lose focus a bit.’ (P3, female, 4th year student, engaged in online for 3 years) ‘If the lights weren’t on at that particular time of having maybe a lecture, then you wouldn’t be able to attend the lecture and even then, you miss out on certain things, you miss out on asking your questions.’ (P6, female, 4th year student, engaged in online for 3 years) ‘Sometimes I always get kicked out of Ulwazi for no reason. I think it has happened a few times … Sometimes that Big Blue button conference tab like it always used to kick you out and then the lecturers wouldn’t see your questions.’ (P8, female, 2nd year student, engaged in online for 1 year)
Unprepared for clinical practicals	‘So practically that was affected, and I feel like coming into 3rd and 4th year I didn’t feel prepared, and I wasn’t. I didn’t trust myself enough with patients.’ (P1, female, 4th year student, engaged in online for 3 years) ‘I don’t think it really helped with clinicals. To be honest we were given very vague PowerPoints and even when we had lectures, it was literally just a lecturer asking us if we have questions, there was very little information that was given especially last year when we were learning about stuff like pathologies and that. So, I think online really didn’t really do a lot for our clinicals. I don’t think it prepared us as well as I think it should have, before we were on campus, where we probably would have gotten a lot more information.’ (P8, female, 2nd year student, engaged in online for 1 year)

### Learning spaces and information communication technology used for online learning

Table 2 indicates that participants could engage in online learning effectively at home but learning at home may have led to a lack of motivation. Furthermore, participants had access to and used computers, smartphones, and the internet for online learning.

### Benefits of online learning

As reflected in Table 3, online learning allowed participants to spend more time learning and less time travelling to the university to attend lectures. Furthermore, online learning was overall cost-effective and less tiring for participants, as they did not need to travel to attend lectures. Learning online also allowed participants to learn in their own spaces, at their own pace, at times that were suitable to them, and learn more independently (Table 3).

### Challenges with online learning

As indicated in Table 4, participants experienced challenges with online learning. They reportedly had limited input from instructors. They also encountered connectivity issues because of load-shedding and had difficulty maintaining a stable connection to LMS, which impacted their interactions with course content and lecturers. Participants also had difficulty managing their time. Learning online also affected participants getting to know their peers and it impacted their preparedness for clinical practicals.

### Strategies employed to facilitate online learning

As reflected in Table 5, participants used these strategies as facilitators for online learning: self-regulated learning, consulting additional resources, instructor presence, provision of internet data bundles, integrated asynchronous and synchronous modes of learning and using a blended approach to online learning.

**TABLE 5:** Strategies employed to facilitate online learning.

Strategy	Interview excerpt
Self-regulated learning	‘I try to stick to like a routine where I wake up at a certain time.’ (P1, female, 4th year student, engaged in online for 3 years) ‘I think I tried to consistently and constantly remind myself that things haven’t changed just because we’re online doesn’t mean the knowledge system has changed. You still need to know this work and you’re still going to need it in practice someday. So, I reminded myself that, everything might be different, you might not be in the lecture hall for tomorrow’s exam, but you need to know this. So, I would sit down, like with every other exam in the past and just prepare, try to understand the work as much as possible, try to simplify it for myself, try to think of possible questions we would be posed the next day.’ (P4, female, 4th year student, engaged in online for 3 years) ‘I think my strategies in that was actually to calm myself down because the frustration of not being able to do, what you need to do, when you need to do it was like the biggest challenge for me and I would get like a really angry very quickly. So, I would actually first have to calm myself down and then troubleshoot my way through it.’ (P6, female, 4th year student, engaged in online for 3 years)
Consulting supplementary material	‘I even YouTubed one or two things that I just did not understand … I actually Googled and YouTube stuff and tried to like watch videos on it.’ (P2, female, 4th year student, engaged in online for 3 years) ‘Another thing that I did was probably, I use a lot of online videos like YouTube and Quizlet and all those kind of stuff too, so online platforms to help my online learning become a bit more effective like Khan Academy and all that kind of stuff. That was like really helpful.’ (P8, female, 2nd year student, engaged in online for 1 year)
Instructor presence	‘But I think with regards to recordings access, speaking to them, communicating them where they be in a meeting or over e-mail, that option was there and the response time is quite great in the sense that maybe they won’t respond the same day, but within the week you will get a response and they are quite willing to help. I think the audio department did well in that sense.’ (P1, female, 4th year student, engaged in online for 3 years) ‘The lecturers were there, they did give support, they even made themselves available for one-on-one sessions, we just, we barely ended up using that option.’ (P9, male, 3rd year student, engaged in online for 2 years)
Provision of internet data bundles for free accommodations	‘I did use the [*name of the University*] data which did help me … I think like [*name of the University*] did try to account for the whole COVID-19 situation as they had given us like an extended break when the outbreak happened. So, I think that helped prepare the lectures and the department on how to prepare the notes and what we needed to learn.’ (P3, female, 4th year student, engaged in online for 3 years) ‘They did try with you know giving us data, so we can access, and we can have data for online learning. … But the university was quite accommodating with that, so they would give us extended amounts of time to do things because they were considerate of the load shedding.’ (P9, male, 3rd year student, engaged in online for 2 years)
Combining asynchronous and synchronous modes of learning	‘So, the recorded lectures is nice to hear because I can go through it at my own pace, absorb it at that point, that’s like beneficial to me, but I do enjoy the live lectures because the difficulty came forgetting questions when it came to the lecture explaining certain concepts, you could ask your question. You would remember like I’m struggling with this concept. So, I personally think a blend of both is important.’ (P1, female, 4th year student, engaged in online for 3 years) ‘I think a combination is good because I sometimes think like the pre-recorded stuff you’re not actively engaging with it as that as you would in a live lecture, but it can be nice to sort of supplement what is said in the live lecture or the other way around. It also allows you to ask questions in the live lecture.’ (P7, female, 3rd year student, engaged in online for 2 years)
Blended approach to online learning	‘I think the blended approach is in my opinion probably better. It’s that sort of intermediate space, right? So, we have online learning and then in person. This is like perfect in terms of it gives you space to do what you have to do in your personal life while also keeping you focused, keeping you aligned with what’s happening. I’m up to date because then you have a few days where it’s you and your laptop where you have to be focused and disciplined but then you also have a few days where you’re reminded that you’re in the midst of all this work where you meet other people. So, in my opinion, if I’m to compare in terms of which is better, I think the blended approach has been really helpful in just giving me space to breathe at times.’ (P4, female, 4th year student, engaged in online for 3 years) ‘I would prefer the blended learning. As I said, you get to have like the best of both worlds. You get to have the discussions and you get to have the recordings of certain things that are important, The in-person lessons, they give you the chance to ask your questions and actually get an answer that isn’t differing from what you ask. Most lecturers make it easier once they also see you, also when they answer questions, others would just answer your question in the most platonic way.’ (P6, female, 4th year student, engaged in online for 3 years)

## Discussion

This qualitative study has provided insights into the online learning experiences of audiology students at a South African university. From the students’ experiences, online learning was reported to be beneficial, but it was not short of challenges, which were compensated for by using strategies for online learning. In addition, it seems that all students had similar online learning experiences regardless of the differences in their demographics.

### Learning spaces and information communication technology infrastructure used for online learning

As implied by the students’ responses, it seems that when engaging in online learning they did so at their homes. The students indicated that their home environment is an optimal learning space for online learning, the space enabled effective and efficient learning at home, thus providing a conducive infrastructure that facilitated online learning. Our result was consistent with Gao et al. (2021) findings. Nevertheless, our finding seems to differ from those reported by Laher et al. (2021) who found that students had difficulties engaging in online learning at home because of having multiple responsibilities at home, such as family responsibilities, which resulted in a lack of motivation to engage in online learning. The participants in our study also reported a lack of motivation but theirs stemmed from the repetitive nature of the course setup for online learning. Our findings and those from Laher et al. (2021) emphasise differences in experiences among students who attended the same university and highlight the need to incorporate accommodations for diverse student needs during infrastructure planning for online learning (Bharuthram & Kies, 2013; Mupawose et al., 2021).

### Benefits of online learning

Online learning as a pedagogical approach requires an efficient information communication technology (ICT) infrastructure to ensure quality learning and teaching and to improve students’ experiences of learning. Similar to the audiology students who participated in Kim’s (2021) study, our study participants favoured engaging in online learning at home because this was a more time and cost-effective mode of learning that offered more learning opportunities. As a result, the participants found online learning to be less exhausting than in-person learning sessions and were thus able to better prepare for assessments and organise their days efficiently. This finding may elaborate on the advantages of online learning (Gajura, 2021).

Online learning also seemed to allow for flexible students’ learning. The students were able to learn at their own pace, unpressurised, and at times that were most suitable to them. Therefore, these results support the findings obtained by Bączek et al. (2021) who also found that students deemed online learning to be advantageous as they were able to learn at their own pace and under comfortable conditions. Furthermore, our study findings and those from Bączek et al.’s (2021) study may be integrated into the expanded description of flexible learning, which supports the notion of providing students with the choice of where and when to learn (Lee & Hannafin, 2016). In addition, flexible online learning encourages self-discipline (Bączek et al. 2021), which are potential attributes that support students’ independence in their learning (Winne & Marzouk, 2019). Therefore, the benefits of online learning bode well with higher-order thinking necessary for a self-regulated who is motivated to improve their learning experiences.

### Challenges with online learning

However, challenges associated with time management during online learning were noticed. Participants in our study found online learning to be challenging as they had to manage their own study time, which contradicts the benefit of time effectiveness as reported by the same participants. The participants attributed their difficulties with time management to procrastination, which is associated with planning or lack thereof. According to Laher et al. (2021) and Rawashdeh et al. (2021), the authors suggested that increased workloads, difficulties structuring the day, multiple responsibilities, a lack of concentration, and unstructured lesson plans consequently lead to poor time management, particularly when engaging in online learning at home (Rawashdeh et al., 2021; Laher et al., 2021). Therefore, the flexibility and independence brought by the online learning approach may not always result in improved preparedness and planning even with adequate infrastructure to support online learning experiences for students.

The active presence of a course instructor aids in students’ active engagement with the course content and allows for student-instructor engagement (Kim et al., 2021). However, some of our participants reported a seemingly limited experience with their course instructors. Laher et al. (2021) found that limited instructor presence resulted in students not understanding the course content. Our findings supported Laher et al.’s (2021) findings, as the students seemed to struggle to engage with the course content in the absence of the course instructors. Furthermore, students reported limited opportunities for peer interactions during online learning, these seemed to be restricted to course-related work, with few opportunities to get to know their peers or form friendships. Laher et al. (2021) found the same among undergraduate psychology students and concluded that this limited peer interaction may lead to students experiencing isolation. Thus, limited instructor presence and peer-to-peer interactions were barriers to building a sense of community among students to encourage collaborative work with the hope of improving students’ experiences with online learning.

Encountering connectivity issues also resulted in online learning being challenging for the students. Our study participants reported that load shedding led to unstable internet connection and failure to power up their devices; similar findings have been reported previously (Azionya & Nhedzi, 2021; Barber & Sher, 2022; Laher et al., 2021). Students also reported experiencing connectivity issues to the LMS and ICT; these disrupted their focus and limited their engagement with synchronous online learning activities, which may have resulted in them missing out on learning opportunities.

Students also felt unprepared for clinical practicals when engaging in online learning, a similar finding was reported by audiology students studying in the United States of America and Turkey (Ölçek et al., 2021; Thome et al., 2020). The students whose responses are reflected in our study seemed to attribute their lack of preparedness for clinical practicals to the limited understanding of theoretical course content. In their strategies for improved clinical training, the participants in our study alluded to in-person clinical training being the most effective means to develop clinical competency, which is similar to the findings obtained by Thome et al. (2020). Seventy-two per cent of the audiology students who participated in the study conducted by Thome et al. (2020) in their survey reported that in-person clinical training was more effective than receiving clinical training through simulations and telepractice. However, traditional in-person clinical training and simulations were complementary learning approaches that improved clinical competency and students’ clinical training experience (Nagdee et al., 2022).

### Strategies employed to facilitate online learning

To facilitate online learning and possibly reap the benefits and overcome the challenges experienced with online learning, the students seemed to have employed strategies that were centred around self-regulated learning (SRL) (Dörrenbächer & Perels, 2016; Panadero, 2017). The participants reported that the following strategies such as consulting supplementary material, instructor presence, and provision of internet data bundles for free and accommodations, combining synchronous and asynchronous modes of learning, and blended learning improved their online learning experiences. Furthermore, Tugtekin (2022) and Barber and Sher (2022) suggested that these strategies were commonly employed by students in higher education to facilitate their ability to manage and comprehend extensive coursework. This was observed during online learning when students engaged independently with content and consulted supplementary materials such as watching videos to increase their learning (Kim et al., 2021). Similar findings were reported by Queiros and De Villiers (2016) that supplementing course material with video clips, allowed students to better understand the course content. In our findings, students reported that the application of SRL strategies, which allowed them to own their learning and accommodate variations in students’ learning strategies. However, theories and models of SRL were not integrated into our study; thus, there is a need to consider these in future research.

A combination of synchronous and asynchronous modes of learning is student-centred and encourages students’ engagement with learning (Lee & Hannafin, 2016). According to the students’ reports in our findings, the use of multiple modes of learning that supplement and complement each other provided them with opportunities to reinforce their understanding of the course content and improve their learning experience. This finding differs from the findings obtained by Singh et al. (2022) who found that when using synchronous and asynchronous modes of online learning audiology students experienced online learning fatigue. However, Singh et al. (2022) attributed this to students’ varying educational levels and their limited experience with online learning. In addition and supporting Singh et al. (2022); and Kim et al. (2021) findings, the students in our study preferred the blended learning approach (Dziuban et al., 2018), which allowed for flexible and independent learning and provided students with opportunities to engage with the course content and co-create with their peers. Our findings highlighted the need to explore the application of student-centred learning theories and practices in online learning for students registered in audiology programmes.

### Study’s strengths

There is a growing interest in understanding students’ experiences as independent life-long learners at institutions of higher learning in South Africa; thus, this study presents findings from students at different educational levels. In addition, the qualitative interviews conducted with the students provided a deeper insight into students’ experiences with online learning. Furthermore, this study employed member checking to ensure participants’ experiences were reflected accurately and truthfully.

### Study’s limitations

Our use of qualitative interviews as the only data collection tool possibly restricted our findings. While the themes of this study managed to explore and understand the audiology students’ experiences of online learning, only one site was used. Yet, it would be beneficial to understand the experiences of audiology students from other universities across South Africa and to also include postgraduate students registered in the same programme.

## Conclusion

The experiences of audiology students who engaged with online learning indicated that most students’ homes were spaces of learning that offered a supportive environment for learning. Online learning offered nuanced benefits that encouraged students to apply self-regulated strategies and to own their learning. The flexibility and cost-effectiveness aspects associated with online learning were favoured by the students and bode well with online learning as facilitators for independent learners. The themes that emerged from this research study provide important insight into online learning approaches, although integrated informally by the students, but were used to improve SRL and student-centred learning and highlighted audiology students’ experiences with online learning. While students reported challenges with online learning such as connectivity issues, load shedding and difficulty managing their time, these were outweighed by the reported benefits, which essentially resulted in overall improved experiences and motivation to use online learning. Finally, our findings provided evidence-based knowledge on the audiology students’ experiences with online learning which may be used to guide curriculum planning for programmes in a South African context.

## Appendix 1: Interview guide

1. Tell me about your experiences with online learning at Wits.
2. Tell me about your experiences with online learning in comparison with in-person learning and blended learning.
3. How was online learning beneficial to you? If any, what were the challenges that you may have experienced with online learning?
4. If at all, how has online learning influenced your clinical practical experience?
5. Please describe your experiences with online learning during the COVID-19 pandemic.
6. What support would you like with engaging with online learning? – Who should provide this support and why?
7. Please tell me about your experiences with engaging with your work.
8. Tell me about the mode of delivery of your work. – Do you prefer live lectures or recorded lectures, and why?
9. Tell me about the strategies you employed when you were engaging with online learning.
10. What changes would you make to online learning at Wits?
